# The Evolution of Nutrition Policy in South Korea: From Aid Recipient to Global Nutrition Policy Model

**DOI:** 10.3390/nu18121959

**Published:** 2026-06-17

**Authors:** Seung Yeon Baek, Young Eun Lee, Ae Rang Lee, Ji-Yun Hwang, Jaehan Kim

**Affiliations:** 1 Department of Food and Nutrition, Chungnam National University, Daejeon 34134, Republic of Korea; yeoni@cnu.ac.kr; 2 Department of Food and Nutrition, Wonkwang University, Iksan 54538, Republic of Korea; 3 Department of Food and Nutrition, Soongeui Women’s College, Seoul 04628, Republic of Korea; 4 Major of Foodservice Management and Nutrition, Sangmyung University, Seoul 03016, Republic of Korea; 5 PAK-Korea Nutrition Center, Faculty of Food, Nutrition and Home Science, University of Agriculture Faisalabad, Faisalabad 38000, Pakistan

**Keywords:** South Korea, nutrition policy, nutrition education, Official Development Assistance, nutrition transition

## Abstract

**Background/Objectives:** South Korea has experienced a rapid transition from widespread food insecurity and undernutrition to a comprehensive and institutionalized nutrition policy system. This study aimed to examine the historical evolution of Korean nutrition policy and nutrition education from the 1960s to the present and to explore its implications for global nutrition governance and nutrition-related Official Development Assistance (ODA). **Methods:** A narrative review was conducted using historical documents, government reports, nutrition policies, national health plans, legislation, and previous academic studies related to Korean nutrition policy and nutrition education. **Results:** Korean nutrition policy evolved through several developmental phases, including an aid-dependent relief period, a state-led food security and school feeding expansion phase, a preventive health and nutrition education phase, and a stage of legal and institutional consolidation. More recently, policies have shifted toward evidence-based, equity-oriented, and life-course approaches. Korea has also expanded its nutrition policy experience through ODA initiatives by supporting institutional development, workforce training, community-based nutrition education, and adaptable nutrition management systems in developing countries. **Conclusions:** Korea’s experience demonstrates how long-term governmental commitment, legislation, surveillance systems, and nutrition education can contribute to national nutrition improvement during rapid socioeconomic transition. These findings may provide useful insights for countries facing the double burden of malnutrition and seeking sustainable and adaptive nutrition policy systems.

## 1. Introduction

As of 2024, the global population has reached approximately 8.2 billion, and advances in economic growth, science, and artificial intelligence (AI) technologies have contributed to unprecedented improvements in human living conditions and convenience [1]. Nevertheless, the double burden of malnutrition, characterized by the coexistence of undernutrition and overnutrition, remains a major global public health challenge. According to a joint report by United Nations Children’s Fund (UNICEF), the World Health Organization (WHO), and the World Bank, approximately 150.2 million children under five years of age suffer from stunting, 42.8 million from wasting, and 35.5 million are overweight [2]. Although the prevalence of stunting has gradually declined over the past decade, approximately 23.2% of children under five worldwide remained affected in 2024, with the majority concentrated in Asia (51%) and Africa (43%) [3]. Despite continuous efforts by national governments and international organizations, including the Food and Agriculture Organization (FAO), UNICEF, and the World Bank, global nutrition problems remain difficult to resolve because of complex factors such as armed conflict, climate change, and economic instability [4,5].

Following liberation from Japanese colonial rule in 1945, the Republic of Korea received approximately USD 12.7 billion in foreign aid until the late 1990s [6]. This assistance included emergency relief, material support, technical cooperation, and economic aid, which played important roles in national reconstruction and socioeconomic development [7,8]. Korea is now recognized as a representative example of a country that successfully transitioned from an aid recipient to a donor country in international development cooperation [7].

In particular, Korea experienced severe socioeconomic collapse after the Korean War (1950–1953), but achieved rapid economic growth beginning with the First Five-Year Economic Development Plan established in 1962 [8]. During this process, Korea became a representative example of the “nutrition transition” model proposed by Popkin [9,10,11]. Unlike many developing countries, Korea experienced rapid economic growth simultaneously with major changes in food supply systems and dietary patterns. Following periods of poverty, famine, and undernutrition during Japanese colonial rule and the Korean War, improvements in food accessibility and the westernization of dietary patterns led to increased energy and fat intake, accompanied by rising rates of obesity, dietary imbalance, and chronic diseases [12,13].

These social and epidemiological changes required substantial shifts in national nutrition policy. Over the past six decades, Korean nutrition policy and nutrition education have continuously evolved in response to economic development, nutrition transition, epidemiological transition, and changing public health priorities [14]. Korean nutrition policy initially focused on food security and the prevention of undernutrition, later expanded toward chronic disease prevention and health behavior modification, and more recently has incorporated digital technologies and personalized nutrition approaches [10,13]. These developments demonstrate that Korean nutrition policy has evolved beyond food provision and nutrition education alone to include legislation, national surveillance systems, life-course nutrition services, interventions for vulnerable populations, and international development cooperation initiatives.

Therefore, the purpose of this study was to examine the chronological evolution of Korean nutrition policy and nutrition education from the 1960s to the present. This study also aimed to identify major laws, national programs, and sociocultural factors that shaped Korean nutrition policy, and to explore the implications of Korea’s experience as a country that transitioned from an aid recipient to a donor country. Furthermore, this study discusses how the Korean experience may provide policy implications for countries facing the double burden of malnutrition and suggests future directions for nutrition policy, including precision nutrition, digital health technologies, health equity, and international cooperation.

## 2. Methods

This study systematically examined the historical development of nutrition policy and nutrition education in South Korea through a narrative review and historical policy analysis approach based on previously published literature and policy documents. Because the primary objective of this study was to examine the long-term evolution and policy transition of nutrition policy and nutrition education rather than to quantitatively evaluate the effectiveness of individual interventions, a narrative review combined with historical policy analysis was considered the most appropriate methodological approach.

**Figure 1:**
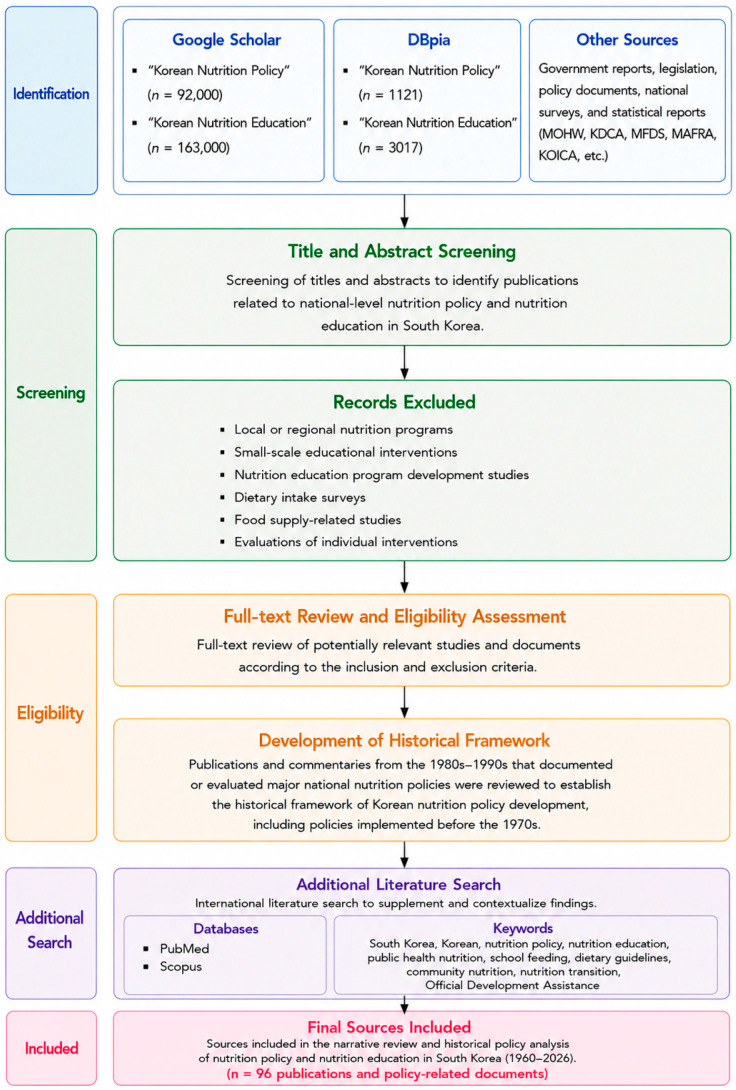
Flow chart of the literature search and selection process for reviewing the evolution of nutrition policy in South Korea.

The literature selection and review process used in this study was summarized in Figure 1. The literature search included publications from 1960 to 2026 and was conducted using both domestic and international databases. Korean-language literature was first searched using Google Scholar and DBpia, a major Korean academic database containing journal articles, dissertations, and government-related publications. Search terms included “한국 영양정책 (Korean-language; Korean nutrition policy)”, “한국 영양교육 (Korean-language; Korean nutrition education)”. A broader search using Google Scholar yielded approximately 92,000 records for Korean nutrition policy and 163,000 records for Korean nutrition education. In DBpia, 1121 records related to Korean nutrition policy and 3017 records related to Korean nutrition education were identified.

Titles and abstracts were screened to identify publications relevant to the historical development of national-level nutrition policy and nutrition education in South Korea. Studies focusing primarily on local or regional nutrition programs, small-scale educational interventions, nutrition education program development, dietary intake surveys, food supply issues, and evaluations of individual nutrition education interventions were excluded. Priority was given to publications addressing policies that were implemented, managed, or evaluated at the national level.

Because relatively few English-language publications addressing Korean nutrition policies were available during the 1970s–1990s, particular reliance was placed on Korean-language academic literature, government publications, policy reports, and commentaries published during this period. Publications and commentaries published during the 1980s and 1990s that documented or evaluated major national nutrition policies were used to establish the historical framework of Korean nutrition policy development, including policies implemented before the 1970s. Based on this framework, the subsequent evolution of Korean nutrition policies and nutrition education programs was examined across different historical periods.

To obtain comprehensive historical and institutional information, government websites, policy reports, legislation, national nutrition surveys, and statistical reports were also reviewed. Sources included documents from major Korean governmental and public institutions, including the Ministry of Government Legislation, Ministry of Health and Welfare, Korea Disease Control and Prevention Agency, Ministry of Food and Drug Safety, Ministry of Agriculture, Food and Rural Affairs, and Korea International Cooperation Agency (KOICA). Reports from international organizations, including the World Health Organization (WHO), Food and Agriculture Organization (FAO), and UNICEF, were also reviewed to interpret the development of Korean nutrition policy within the broader context of global nutrition governance and international development cooperation.

Additional literature searches were conducted using PubMed and Scopus with the keywords South Korea, Korea, nutrition policy, nutrition education, public health nutrition, school feeding, dietary guidelines, community nutrition, nutrition transition, and Official Development Assistance. These international sources were used to supplement, verify, and contextualize the historical findings derived from domestic literature.

Following title and abstract screening, application of the inclusion and exclusion criteria, and full-text review, a total of 96 publications and policy-related documents were ultimately included in the narrative review and historical policy analysis.

Data analysis was conducted using a chronological approach. Major policy phases were identified according to socioeconomic changes, epidemiological transition, and shifts in public health priorities. For each period, policy characteristics, institutional changes, development of nutrition programs, target populations, implementing organizations, legal frameworks, and policy objectives were analyzed to identify the developmental patterns of Korean nutrition policy. Based on these findings, the evolution of Korean nutrition policy was interpreted within the broader framework of aid dependency, state-led expansion, preventive health transition, institutionalization, evidence-based policy development, equity-oriented approaches, and expansion into international development cooperation.

## 3. Before the 1970s

Before the 1970s, Korean nutrition policy was characterized as an “aid-dependent phase” based largely on international assistance and technical support from global organizations. Although a self-sustaining national nutrition policy system had not yet been fully established during this period, it represented an important foundational stage that contributed to the development of later state-led nutrition policies through the establishment of institutional and human resource infrastructures.

### 3.1. Nutrition Relief Activities of International Organizations and Private Relief Agencies

The World Health Organization (WHO) established an office in Korea in 1962 and provided various technical and institutional forms of support to improve public health and nutrition [15]. WHO’s activities during this period extended beyond nutrition policy and included capacity building for public health professionals, as well as support for disease control programs related to tuberculosis, parasitic diseases, and maternal and child health. In 1968, WHO collaborated with UNICEF and the Food and Agriculture Organization (FAO) to implement the Applied Nutrition Project (ANP), primarily targeting rural communities. This initiative introduced an early community-based nutrition intervention model integrating nutrition education, dietary improvement, and local workforce training [15,16].

UNICEF began its official activities in Korea in 1948 [17]. Initially, UNICEF focused on tuberculosis prevention through Bacillus Calmette–Guérin (BCG) vaccination programs and supported domestic vaccine production by providing equipment for BCG and Diphtheria, Pertussis, and Tetanus (DPT) vaccine manufacturing [17]. In response to requests from the Korean government in 1953, UNICEF also distributed milk powder to nutritionally vulnerable populations, including children without regular meals, infants, and pregnant women, thereby contributing substantially to postwar nutritional support [17,18]. Between 1954 and 1957, UNICEF supplied milk powder to approximately 1.5 million children through a nationwide school milk feeding program, which became one of Korea’s earliest national nutrition improvement initiatives [18]. This program later influenced the ANP approach by integrating nutrition education, school feeding, and community organization. As part of the ANP, UNICEF also promoted leadership training in nutrition education and encouraged household consumption and local production of nutritious foods [18].

FAO dispatched a technical mission to Korea in 1952 to support the establishment of the “Five-Year Agricultural, Forestry, and Fisheries Reconstruction and Development Plan” and to provide technical consultation for improving agricultural productivity and food self-sufficiency [13]. From 1962 to 1980, FAO also participated in the establishment and revision of the Korea Recommended Dietary Allowances (KRDA), contributing to the development of national nutrition standards and policies [19,20]. However, before the 1970s, national nutrition standards were developed despite the absence of comprehensive nationwide dietary intake surveys. Consequently, the standards were largely based on international nutritional recommendations, East Asian or Japanese reference data, and limited domestic dietary intake studies [13,21]. National nutrition surveys in Korea were conducted under the supervision of the Ministry of Health and Social Affairs, and the first nationwide nutrition survey was performed in 1969 [21]. Because a complete population survey was not feasible, 952 households from Seoul, Gyeonggi Province, and Chungcheong Provinces were selected for a 32-day survey to evaluate dietary intake and nutritional status among the Korean population [21,22].

CARE (Cooperative for American Relief Everywhere), one of the major American private relief organizations established after World War II, entered Korea in 1949 following the completion of emergency relief operations in Europe [23]. Although CARE temporarily withdrew during the Korean War, it later resumed relief activities under the supervision of the United Nations Civil Assistance Command, Korea (UNCACK) [23]. CARE became one of the largest food donors in Korea and implemented the Surplus Utilization for Nutrition (SUN) program, supplying grains, infant foods, and canned products [23]. Following the Korean War, CARE also supported the distribution of surplus agricultural commodities from the United States under Public Law 480, also known as the Agricultural Trade Development and Assistance Act of 1954 [23,24]. CARE additionally operated childcare and feeding programs for children throughout Korea and employed 15 nutritionists who were assigned to regional centers to conduct village-based nutrition education programs [23]. Beginning in 1957, after the completion of UNICEF’s official milk feeding project, CARE assumed responsibility for continuing milk distribution and corn-based school lunch programs for elementary school students [18,23,25]. CARE also conducted nutrition promotion campaigns using calendars, booklets, comics, and posters as educational media [26]. In 1970, the organization broadcast short nutrition education messages through the Korean Broadcasting System (KBS) radio network, representing one of the earliest attempts to use mass media for nutrition education in Korea [27]. Furthermore, with support from the Ministry of Health and Social Affairs and WHO, CARE published educational materials such as What Shall We Eat? (1975) and Let Us Stay Healthy (1977) as part of integrated dietary improvement and family planning programs [28].

### 3.2. Domestic Nutrition-Related Policies

#### 3.2.1. Applied Nutrition Project

Before the 1960s, nutrition-related research and activities in Korea were largely academic and laboratory-oriented, with limited practical application and policy integration. Beginning in the 1960s, however, the introduction of the Applied Nutrition Project (ANP), led by the Rural Development Administration with support from international organizations, marked a transition toward community-based nutrition policies and education programs [16,17]. The ANP was a comprehensive rural nutrition improvement initiative that integrated demonstration education, school feeding, food production, and cooking skills training to improve dietary practices, nutritional status, and health conditions in rural communities [16,17].

For children, the ANP developed nutrition education, supplementary feeding, maternal nutrition support, and income-generating programs to improve growth and development among rural populations. One or two villages in each province were selected as pilot communities for implementation [16]. The project also provided rural women with training in cooking techniques and food processing methods and promoted balanced diets and institutional foodservice practices through menu development using local ingredients, group foodservice education, and hygiene training [16]. To reach geographically isolated mountain and rural communities, mobile nutrition education vehicles equipped with audiovisual devices and cooking demonstration tools were operated using vehicles donated by FAO and WHO [16]. In addition, training and capacity-building programs for field workers were implemented to establish a workforce capable of conducting future community nutrition programs [16].

The Applied Nutrition Program implemented in rural Korea during the late 1960s and 1970s represented one of the earliest community-based nutrition interventions in the country [29]. The program was introduced with technical and financial support from UNICEF, FAO, and WHO and aimed not only to improve nutritional status but also to enhance food production, nutrition education, and community participation [15,16,18,29]. The emergence of the program should be understood within the broader context of post-war reconstruction, rapid population growth, persistent food shortages, and the government’s developmental-state strategy during the early stages of economic development. At the time, improving nutritional status was regarded not only as a public health objective but also as an essential component of rural development, labor productivity, and national modernization. Implemented through demonstration villages and integrated with broader rural development initiatives, the program reflected the government’s recognition that nutrition improvement was closely linked to agricultural development, public health, and socioeconomic modernization [29]. Furthermore, by combining nutrition education with food production, food utilization, and community participation, the program established an integrated approach to nutrition improvement that later aligned with the objectives of rural modernization and the Saemaul Movement [29]. The program contributed to improvements in dietary diversity, nutrient intake, child growth, and nutrition-related health outcomes, while also establishing an institutional foundation for subsequent community nutrition programs in Korea [29].

#### 3.2.2. School Foodservice Before the 1970s 

School foodservice in Korea began in 1953 and was initially characterized as a relief feeding program for elementary school children based on food aid provided by international organizations [30]. The earliest school meals consisted primarily of bread made from donated wheat or corn flour and soybean milk prepared for children experiencing food insecurity [17,30]. Before the 1960s, bread-based meals were the dominant form of school feeding; however, beginning in the 1960s, the system gradually transitioned from relief-oriented feeding to fee-based foodservice supported by domestic administrative systems [30].

Following the withdrawal of foreign relief organizations in 1966, the Ministry of Education inherited the administrative networks, feeding management personnel, and food supply systems previously established by international organizations and utilized them as the foundation for a national school foodservice administration system [30]. This transition contributed to the development of school foodservice as a national policy aimed not only at supporting food-insecure children but also at improving student nutrition and promoting dietary education. School meals increasingly emphasized nutritionally balanced diets that reflected Korean dietary preferences and encouraged healthier eating behaviors [17,18].

#### 3.2.3. The Dietitian System

The introduction and expansion of the dietitian system represented a critical foundation for the implementation of Korean nutrition policy. The legal basis for the dietitian profession was established through the Food Sanitation Act enacted in 1962, and the national licensing system was introduced shortly thereafter, with the first dietitian licenses issued in 1964 [16,31]. The establishment of the profession during a period still heavily dependent on international aid was particularly significant because it reflected simultaneous efforts to institutionalize nutrition-related professional manpower. At that time, Korea recognized the need to develop trained professionals capable of systematically managing nutrition issues at the national level while continuing to receive international support to address food shortages and undernutrition [17]. Consequently, the introduction of the dietitian system represented an important transition from a relief-oriented approach toward a self-sustaining nutrition policy system based on professional expertise [16].

During the 1970s and 1980s, the expansion of school foodservice programs further increased the placement of dietitians in public institutions, thereby strengthening the implementation capacity of nutrition policies. Subsequently, the professional roles of dietitians expanded into public health centers, hospitals, industrial foodservice settings, and Centers for Children’s and Social Welfare Foodservice Management [16,31,32]. Through continuous revisions of related legislation and institutional frameworks, the dietitian profession became established as a licensed professional workforce and a key infrastructure component of Korean nutrition policy implementation [15].

## 4. The 1970s

The 1970s represented a period during which Korean nutrition policy became actively promoted under strong state leadership, with a primary focus on food security and public enlightenment. This period can therefore be characterized as the “food security and public education phase.” In particular, nutrition policy became closely integrated with the Saemaul Movement, transforming dietary improvement from individual health education into a nationwide social movement.

### 4.1. Saemaul Movement

The Saemaul Movement included not only economic and environmental improvements in rural communities but also health and nutrition activities aimed at improving dietary practices and nutritional status among the population. This movement is considered an expansion of the community-based nutrition education models and pilot village experiences previously accumulated through the Applied Nutrition Project (ANP) during the 1960s [18]. During the early stages of the Saemaul Movement, the primary focus was on improving rural living conditions and increasing household income. However, after 1973, the movement expanded to include dietary improvement campaigns, rice-saving movements, mixed-grain and flour-based diet promotion campaigns, kitchen modernization projects, nutrition education, cooking demonstrations, and community foodservice activities [33].

One of the representative dietary improvement initiatives of the Saemaul Movement was the promotion of rice-saving and mixed-grain consumption campaigns. Because rice supplies were insufficient at that time, the government encouraged the consumption of mixed grains and flour-based foods at schools, workplaces, and households in order to strengthen national food self-sufficiency [34]. This policy emerged not only from concerns regarding nutrition but also from broader economic and food security challenges, including chronic rice shortages, limited foreign exchange reserves, and increasing dependence on imported grain during a period of rapid industrialization [35]. Therefore, the promotion of mixed-grain consumption was intended primarily to stabilize the national food supply by substituting rice with relatively inexpensive cereals such as barley and wheat [35]. At the same time, these campaigns extended beyond simple food-saving measures and were expected to improve dietary balance and diversify grain consumption patterns, thereby contributing to national nutrition improvement [34]. In addition, the government organized nutrition education and cooking practice sessions through local women’s associations affiliated with the Saemaul Movement and provided education on canning, food drying, and food preservation techniques to improve household food storage capacity and reduce food waste [36]. Kitchen modernization and communal kitchen projects were also promoted to improve household sanitation and living environments [36]. Traditional kitchens were renovated by installing smoke ventilation systems and improving sanitary conditions. Community kitchens additionally served as centers for cooking classes, dietary education, and group foodservice training, thereby strengthening community-based nutrition education [36].

The importance of maternal and child nutrition management was also emphasized during this period. Childcare centers, supplementary feeding programs, and monitoring systems for malnourished children were introduced to protect nutritionally vulnerable populations in rural communities [36]. These dietary and nutrition improvement policies extended beyond simple food supply measures and represented broader national public health strategies aimed at improving nutritional status, modernizing living culture, strengthening community capacity, and institutionalizing nutrition education systems. Ultimately, the nutrition improvement activities implemented during the Saemaul Movement period contributed to the later establishment of the School Meals Act, the introduction of national health promotion policies, and the expansion of community nutrition programs [18].

The emergence of nutrition-related programs during the 1960s and 1970s was closely associated with broader national development objectives. During this period, food security was regarded not only as a public health concern but also as a matter of economic viability, national security, and rural development. As rapid industrialization accelerated under the Five-Year Economic Development Plans, concerns regarding food shortages, increasing dependence on grain imports, and widening rural–urban disparities became major policy issues [29,35].

Consequently, nutrition improvement initiatives were closely integrated with agricultural modernization and rural development efforts, reflecting the government’s developmental-state strategy aimed at simultaneously improving food security, rural livelihoods, and national economic growth [29,35].

### 4.2. National Nutrition Survey

Beginning in 1962, the Ministry of Health and Social Affairs conducted the National Health and Attitude Survey to investigate public awareness of infectious diseases, health behaviors, and nutrition-related attitudes [37,38]. In 1969, the first nationwide National Nutrition Survey was conducted through technical cooperation between the Ministry of Health and Social Affairs and FAO, and nationwide nutrition surveys subsequently became more regularly implemented throughout the 1970s [21].

The National Nutrition Survey collected data using household food weighing methods, 24 h dietary recall interviews, and biochemical indicator analyses. These surveys provided some of the earliest national statistical data on energy, protein, fat, calcium, and vitamin intake levels, as well as the nutritional status of the Korean population [21].

Throughout the 1970s, the survey results served as an important evidence base for national nutrition and dietary policies. In particular, the findings were used to support and justify policies related to mixed-grain consumption campaigns, rice-saving initiatives, restructuring of grain consumption patterns, expansion of school foodservice, and rural lifestyle improvement programs [34]. This survey-based approach marked an important transition in Korean nutrition policy from aid- and supply-oriented interventions toward evidence-based policymaking grounded in national statistics and surveillance systems.

In addition, beginning in the late 1970s, the focus of nutrition and lifestyle research gradually shifted from the simple resolution of nutritional deficiencies toward broader concepts of dietary improvement, prevention, nutrition education, and behavioral change. Consequently, the National Nutrition Survey and related health and attitude surveys conducted during the 1970s established an early foundation for evidence-based nutrition policy and later contributed to the development of national legislation and nutrition management systems, including the National Health Promotion Act of 1995 and the National Nutrition Management Act of 2010.

### 4.3. School Foodservice Since the 1970s

During the 1970s, Korean school foodservice transitioned from the relief-feeding programs of the 1950s and 1960s, which had depended heavily on international aid organizations, toward a national policy framework focused on nutrition and dietary improvement. Following the termination of food assistance from international organizations such as CARE in 1966, the Korean government assumed responsibility for the school foodservice administrative system, and school meals gradually shifted from relief-oriented free or low-cost feeding programs toward fee-based and self-sustaining foodservice systems [18].

During this period, schools functioned as key educational platforms for implementing dietary improvement policies associated with the Saemaul Movement. The government actively promoted rice-saving and mixed-grain consumption campaigns through school-centered activities, including lunchbox inspections, “flour-based food days” twice per week, and increased consumption of mixed grains and wheat products [34]. These initiatives were not merely food-saving campaigns but also educational strategies designed to transform national dietary practices and disseminate national food policies through students and households. At the same time, cooking education, nutrition education, and healthy eating habit formation programs were strengthened, contributing to the development of schools as formal delivery systems for nutrition education.

However, in 1977, a large-scale food poisoning outbreak associated with cream bread distributed in several schools in Seoul exposed weaknesses in hygiene management, food preparation, distribution, and storage systems within school foodservice programs [24,39]. This incident highlighted the need for an institutional management system integrating food safety, hygiene control, nutrition management, and administrative supervision. Subsequently, the School Meals Act was enacted in 1981, establishing national standards for foodservice facilities, nutrition management, and school meal operations [18].

## 5. The 1980s

The 1980s represented a period during which Korean nutrition policy shifted from a primary focus on food security and undernutrition toward prevention-oriented approaches emphasizing healthy behaviors and chronic disease prevention. As the national disease burden transitioned from infectious diseases to chronic noncommunicable diseases, nutrition policy increasingly expanded beyond food provision to promote dietary behavior change and public health improvement.

### 5.1. National Healthy Lifestyle Guidelines

During the 1980s, rapid economic growth and industrialization substantially changed dietary patterns and food consumption behaviors in Korea. Increased household income and urbanization accelerated the growth of the food and restaurant industries, leading to a rapid increase in the consumption of instant foods, processed foods, and fast foods. In particular, preparations for the 1988 Seoul Olympic Games contributed to diversification of the tourism and foodservice industries and accelerated changes in dietary culture and eating environments [40,41].

At the same time, major shifts occurred in the national patterns of mortality and disease. Whereas infectious diseases such as tuberculosis, bronchitis, and other communicable diseases had been the major causes of mortality during the 1950s and 1960s, the prevalence of chronic diseases closely associated with dietary habits and lifestyles—including cerebrovascular disease, hypertension, and cancer—increased substantially beginning in the 1970s. Consequently, the government recognized the need for policy interventions aimed at improving unhealthy dietary practices and promoting healthy lifestyle behaviors [40].

In response to cholera outbreaks and related deaths during the early 1980s, concerns regarding public hygiene standards and health awareness increased nationwide [42]. In July 1984, the Ministry of Health and Social Affairs announced the “National Healthy Lifestyle Guidelines” (Table 1). The guidelines included seven practical recommendations related to personal hygiene, dietary habits, exercise, moderation, and health management, including messages such as “Wash your hands before meals and brush your teeth after meals” and “Eat balanced and less salty meals regularly” [20,43]. This approach later evolved into the four major health promotion practices—smoking cessation, alcohol moderation, physical activity, and nutrition—adopted within the National Health Promotion Plan introduced in 2002 [20,43].

Although nationwide nutrition surveys had been conducted since the 1960s, limitations in survey scale and administrative systems restricted the ability to comprehensively evaluate individual nutritional status and health conditions [43]. Furthermore, earlier nutrition policies had primarily focused on food supply and the prevention of nutritional deficiencies. Therefore, the National Healthy Lifestyle Guidelines represented one of the earliest national-level practical guidelines integrating nutrition with health behavior promotion. This initiative marked an important transition of Korean nutrition policy toward prevention-oriented and behavior-centered approaches [42,44].

### 5.2. Dietary Guidelines for Koreans

Since its establishment in 1967, the Korean Nutrition Society has conducted academic research and provided policy recommendations aimed at improving the nutritional status of Koreans and advancing nutrition science. Following the introduction of the National Healthy Lifestyle Guidelines in 1984, the society published the “Dietary Guidelines for Koreans (Table 2)” based on scientific nutritional evidence, thereby playing a major role in shaping Korean nutrition policy [45].

These guidelines were significant because they translated nutrient intake recommendations into practical dietary behaviors that could be implemented in everyday life rather than simply presenting nutrient intake standards. In addition, the guidelines reflected the growing recognition of chronic disease prevention through dietary improvement, which had become an important public health concern during the 1980s. This approach subsequently influenced the development of national nutrition policies, dietary education programs, and the establishment and revision of dietary reference standards in Korea.

### 5.3. Meal-Ordering System

Prior to the 1988 Seoul Olympic Games, the Ministry of Health and Social Affairs introduced the “customer meal-ordering system” to promote dining practices that aligned with international standards [41]. This policy aimed to reduce excessive multi-side-dish traditional meal settings and encouraged serving side dishes individually using separate tableware. The number of basic side dishes was generally limited to five or fewer, with additional dishes provided only upon customer request. The policy emphasized hygiene, food conservation, simplified meal service, and improved customer service standards. Restaurants were also encouraged to install food display cases to reduce food waste and improve hygiene and service quality [41].

Although the policy was reported to produce positive improvements in some restaurants during the Olympic period, it did not become firmly established over the long term. Traditional Korean dining culture, which favored generous multi-side-dish meal settings, remained deeply rooted in households and restaurants. This case demonstrated that dietary improvement efforts during the 1980s could not be sustainably maintained through state-led campaigns and institutional recommendations alone and highlighted the importance of broader cultural acceptance and structural dietary changes [24,41].

### 5.4. School Meals Act

The 1980s represented a major transitional period during which Korea established the foundation of the modern school foodservice system through nationwide implementation of on-site meal preparation, establishment of nutritional standards, and deployment of professional personnel such as dietitians and cooks [30]. Prior to this period, school meals primarily functioned as nutritional supplementation programs; however, beginning in the 1980s, they evolved into comprehensive meal services prepared and served within schools as part of the formal educational system.

In particular, the enactment of the School Meals Act in 1981 institutionalized school foodservice as a national policy supported by a legal and administrative framework. The Act systematically established standards for foodservice facilities, hygiene management, nutrition management, and operational systems [32]. As a result, school meals expanded beyond nutritional supplementation and increasingly served educational and public health functions aimed at promoting student health and healthy eating habits.

To strengthen foodservice management, dietitians began to be assigned to schools beginning in 1978 and were officially employed as public health professionals [30,32]. In addition, the previous system, which had relied heavily on voluntary parental participation in meal preparation, was replaced by professional staffing systems that assigned foodservice personnel to schools, including one worker in remote rural areas and four workers in urban areas [30]. These developments demonstrated the transition of Korean school foodservice from a relief-oriented supplementary feeding system toward an institutionalized nutrition management system supported by professional personnel and legal standards.

## 6. The 1990s

The 1990s can be characterized as an “institutionalization phase” in which Korean nutrition policy became legally and institutionally established following the enactment of the National Health Promotion Act. During this period, the government formally recognized its responsibility for nutrition management and health promotion, and nutrition policy became established as a central component of public policy rather than a collection of individual projects or campaigns.

### 6.1. National Dietary Guidelines

As rapid economic growth and improvements in living standards accelerated during the 1990s, the prevalence of chronic diseases increased substantially, accompanied by rising healthcare expenditures. Consequently, the need for preventive strategies based on nutritional approaches became increasingly emphasized [44]. During this period, consumption of foods made with wheat flour, sugar, and fats expanded through both international food aid and the growth of the domestic food industry. In addition, the popularity of energy-dense, high-fat, and high-sugar foods such as pizza, hamburgers, fried chicken, and doughnuts increased rapidly [44]. Although the foodservice industry experienced annual growth rates approaching 30%, food industry management policies remained largely focused on food hygiene and sanitation, leading to growing concerns regarding dietary quality and chronic disease prevention [44].

In response, the Korean government consolidated and standardized recommendations previously issued by nutrition-related organizations and introduced the “National Dietary Guidelines” in December 1990 to improve public understanding and practical implementation of healthy dietary behaviors (Table 3) [20,42,46]. The guidelines included detailed behavioral recommendations and represented the first national dietary guidelines in Korea to provide quantitative nutritional recommendations, such as limiting fat intake to approximately 20% of total energy intake [20,42]. In addition, as obesity associated with excessive nutrient intake emerged as a major public health concern, the guidelines incorporated recommendations for maintaining healthy body weight and limiting daily salt intake to less than 10 g [20,42].

**Figure 2:**
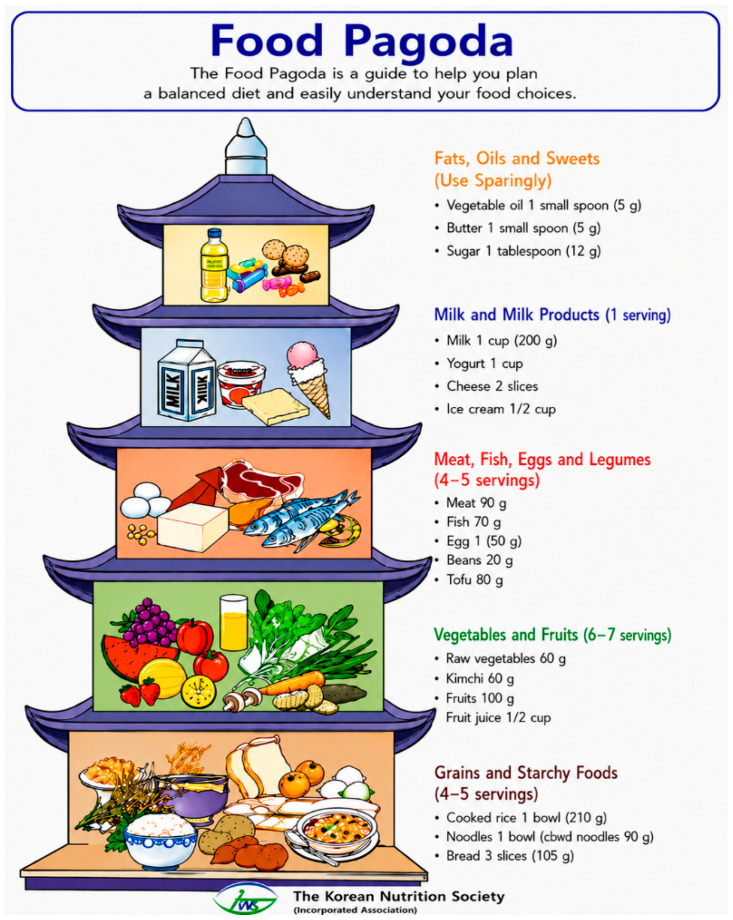
Food Pagoda in Korea (1995). Food Pagoda proposed by The Korean Nutrition Society [47].

However, although the National Dietary Guidelines presented evidence-based nutritional recommendations, they remained somewhat abstract and difficult for the general public to apply directly to daily meal planning. Consequently, the Food Tower (Food Pagoda) was introduced in conjunction with the sixth revision of the Korea Recommended Dietary Allowances (KRDA) in 1995 [20]. The Food Pagoda was designed based on the traditional Korean five-story pagoda structure, with the size and position of each level symbolizing the relative importance and recommended intake of the five food groups classified at that time [47] (Figure 2). The Food Pagoda served as a visual educational tool intended to improve public understanding of dietary guidelines and promote healthy eating patterns [20]. In 2010, the Food Pagoda was later revised into the Food Balance Wheel to further emphasize the importance of physical activity and adequate water intake [48].

### 6.2. National Health Promotion Act

The National Health Promotion Act was passed by the National Assembly in 1994, enacted on 5 January 1995, and implemented beginning on 1 September 1995 [49]. Article 1 of the Act stated that the government should promote proper health knowledge and create conditions that enable citizens to practice healthy lifestyles independently while fostering awareness of personal responsibility for health. Article 15 specifically addressed “nutrition improvement,” stipulating that the national and local governments should investigate the nutritional status of the population, establish nutrition improvement measures, and conduct nutrition guidance, nutrition education, and nutrition-related research programs [49]. In addition, Article 16 provided the legal basis for the National Nutrition Survey, which had been conducted since 1969, by requiring the Ministry of Health and Social Affairs to regularly investigate the population’s health status, food intake, dietary habits, and nutritional conditions [49].

This legislation represented a major turning point because it formally defined the promotion of healthy lifestyles as a legal and institutional responsibility of the state rather than merely a public recommendation. In particular, the Act established “nutrition improvement” as a central component of national health promotion policy and provided a legal framework for nutrition surveys and nutrition education programs, thereby institutionalizing systematic national nutrition policy implementation. Furthermore, the Act contributed to the transformation of nutrition surveys from fragmented assessments into data-based public health administration systems supporting policy development and evaluation.

### 6.3. Korea National Health and Nutrition Examination Survey

Following the enactment of the National Health Promotion Act in 1995, the existing National Nutrition Survey (1969–1995) and the National Health and Health Behavior Survey were integrated into the Korea National Health and Nutrition Examination Survey (KNHANES), which was formally launched in 1998 [50,51]. KNHANES represented a major advancement in national nutrition surveillance because it shifted from household-level food consumption assessments to individual-level investigations and expanded to include comprehensive information on dietary intake, health status, disease prevalence, and health behaviors [51].

Initially conducted every three years, KNHANES utilized increasingly sophisticated sampling designs that improved the representativeness and reliability of national health and nutrition data [52]. This survey system enabled more systematic analysis of the relationships between dietary behaviors and chronic diseases and supported evidence-based policymaking in nutrition and public health [51]. The introduction of KNHANES demonstrated the transition of Korean nutrition policy from simple nutritional status monitoring toward integrated, evidence-based policy systems considering health behaviors and disease-related factors simultaneously [51]. Furthermore, because KNHANES adopted structures similar to those used in international health and nutrition surveys, it provided a foundation for internationally comparing and evaluating the nutritional and health status of the Korean population [51].

Along with the establishment of KNHANES and the strengthening of national nutrition surveillance systems, the role of nutrition policy in Korea was gradually redefined. During the 1990s, Korean nutrition scholars increasingly argued that nutrition policy should expand beyond food supply management and nutrient deficiency prevention to encompass nutrition education, food labeling, social welfare programs, and health promotion initiatives [53]. At the same time, revisions of the Korean Recommended Dietary Allowances (RDAs) gradually shifted their focus from supporting food supply planning and preventing nutrient deficiencies toward chronic disease prevention, avoidance of excessive nutrient intake, and the development of dietary guidelines for healthy populations [53,54]. These developments occurred alongside broader social and political transformations following democratization and the expansion of social welfare institutions in Korea, as well as growing concerns regarding population aging and the increasing burden of chronic diseases [55]. Consequently, nutrition policy increasingly evolved from a food security-oriented approach toward a comprehensive framework emphasizing health promotion, disease prevention, and evidence-based public health interventions [53,54,55].

### 6.4. Pilot Health Promotion and Nutrition Programs at Public Health Centers

Following the enactment of the National Health Promotion Act in 1995, community-based health promotion programs were actively implemented, and pilot nutrition improvement projects were introduced at selected public health centers [56]. These programs included maternal and child nutrition management, nutrition education for pregnant and lactating women, nutrition camps for elementary school students, and chronic disease prevention education programs tailored to different life stages and health conditions [56].

These pilot projects evolved beyond simple nutrition education into practical intervention programs that reflected local health problems and community living environments. They also played an important role in establishing community-based nutrition service delivery systems centered on public health centers [56]. Furthermore, these initiatives provided the policy foundation for later expansion into nationwide integrated health promotion programs and the Nutrition Plus program [56].

This transition indicated a shift away from uniform, centrally directed policies toward community-based nutrition policies tailored to local characteristics and needs. It also established public health centers as key implementation agencies for community nutrition management and health promotion activities [56].

## 7. The 2000s

Nutrition policy in Korea during the 2000s was characterized by a transition toward evidence-based policymaking through the establishment of the National Health Plan and the institutionalization of the Korea National Health and Nutrition Examination Survey (KNHANES). In particular, the expansion of data-driven policy development and nutrition support programs targeting vulnerable populations demonstrated increasing scientific rigor, policy targeting, and health equity within Korean nutrition policy. This period is also notable because universal health promotion strategies and selective nutrition support programs for vulnerable groups were implemented simultaneously, resulting in a more sophisticated national nutrition policy system.

### 7.1. National Health Plan (Health Plan 2010)

The National Health Plan, first established in 2002, was legally based on the National Health Promotion Act enacted in 1995. The plan functioned as a long-term national strategy for health promotion, designed on a 10-year basis and evaluated and revised every five years. Beginning with Health Plan (HP) 2010, the first phase (2002–2005; Table 4) and second phase (2006–2010; Table 5) were implemented during the 2000s. In this study, the nutrition-related components among the major healthy lifestyle practice areas—including smoking cessation, alcohol moderation, nutrition, and physical activity—were primarily examined.

The first National Health Plan focused on correcting nutritional imbalances and promoting healthy dietary habits, whereas the second phase established more specific and measurable objectives. Compared with the first phase, the revised second phase emphasized several major policy directions [57,58]. First, nutrition support programs were developed for nutritionally vulnerable populations, including pregnant women, infants, older adults, individuals with disabilities, and socially disadvantaged children [57,58]. Second, educational materials and practical resources promoting healthy dietary behaviors were developed based on the coexistence of undernutrition and overnutrition and the nutritional characteristics of the Korean population [57,58]. Third, efforts were made to institutionalize nutrition labeling systems for processed foods in response to increasing consumer demand for health management, the need for national nutrition management, and the expansion of food imports and exports [57,58]. Fourth, systems for producing and disseminating national nutrition information were established to support the monitoring and management of population health and nutritional status [57,58]. Fifth, nutrition-dense meal plans, recipes, and cooking methods for older adults were developed in response to rapid population aging [57,58].

Unlike smoking or alcohol consumption, nutrition was recognized as a fundamental determinant of health influencing food choices, dietary behaviors, disease prevention, and health promotion throughout the life course. Consequently, the National Health Plan provided the policy basis for strengthening public nutrition infrastructure, including nutrition counseling services at public health centers, community nutrition education programs, and the Nutrition Plus program [57,58].

The National Health Plan has considerable symbolic and policy significance because it serves as a national roadmap for health promotion and establishes national public health goals and indicators [57,58]. In the field of nutrition, the plan was particularly meaningful because it positioned nutrition policy not merely as an educational activity but as a policy domain guided by national health objectives and measurable performance indicators.

### 7.2. Dietary Goals for Koreans

The “Dietary Goals and Guidelines for Koreans” (Table 6) were established by the Ministry of Health and Welfare through interministerial collaboration between 2002 and 2003 [59]. These goals were developed based on the nutritional intake status, disease patterns, and dietary characteristics of the Korean population. To facilitate implementation, detailed practical guidelines known as the “Dietary Guidelines for Koreans” were also developed [59]. Furthermore, age-specific dietary guidelines were subsequently created and utilized as nutrition education materials in public health centers and hospitals to promote public health and quality of life [59].

These dietary goals were developed in response to rapidly increasing obesity rates and the growing socioeconomic burden of chronic diseases. Accordingly, they emphasized the prevention of excessive energy intake and promoted balanced consumption across food groups as national dietary standards [59]. In particular, the guidelines encouraged the preservation of traditional Korean dietary culture based on vegetable and fermented food consumption, thereby contributing not only to nutritional balance but also to the cultural sustainability of healthy eating patterns [11].

Compared with the National Dietary Guidelines introduced during the 1990s, these dietary goals and guidelines provided more specific and practical behavioral recommendations. Consequently, they represented an important advancement in the development of action-oriented nutrition education materials that could be more easily understood and implemented by the general public.

### 7.3. Nutrition Plus Program

Despite continued economic growth during the early 2000s, nutritional imbalances among low-income pregnant women, lactating women, and infants remained an important public health concern in Korea. In particular, deficiencies in nutrients such as iron, calcium, and vitamins, along with health disparities related to unequal food accessibility, became increasingly recognized as major public health issues. To address these problems, the need emerged for a national nutrition support program integrating supplementary food provision with nutrition management and education rather than relying solely on nutrition education.

Reflecting these policy needs, Korea introduced the Nutrition Plus Program (Supplementary Nutrition Program for Pregnant Women and Infants) as a pilot project in selected regions beginning in 2005, and its effectiveness and operational feasibility were subsequently confirmed through pilot implementation [60]. Following the establishment of institutional and administrative systems, the program was expanded nationwide in 2008 as a government-led initiative under the Ministry of Health and Welfare [60].

During the design and development process, Korea used the United States Special Supplemental Nutrition Program for Women, Infants, and Children (WIC) as a major benchmarking model [61]. The WIC program provides supplementary foods, nutrition education, and linked health services for low-income pregnant women, lactating women, and infants and has been recognized as an effective intervention for improving nutritional status and reducing health disparities [62]. Accordingly, Korea conducted a series of studies analyzing the structure and operation of the WIC program in order to develop an appropriate nutrition support program adapted to domestic conditions [60,61,62]. Initial studies proposed strategies for establishing a national nutrition assistance program targeting nutritionally at-risk populations based on the WIC model, thereby establishing the conceptual foundation of the Nutrition Plus Program [60,61,62]. Subsequent pilot projects evaluated the applicability and effectiveness of core components, including supplementary food provision, nutrition education, and individualized counseling [57,58,59]. Furthermore, implementation strategies and expansion plans were developed to support nationwide program dissemination and institutionalization [60,61,62].

Although the Nutrition Plus Program adopted an integrated approach similar to the WIC program by combining supplementary food support with nutrition education, the Korean model differed by utilizing a public health center-based service delivery system for participant management and community-based services. In addition, participant selection involved not only income criteria but also nutritional risk assessments, enabling more targeted support based on nutritional need.

Evaluation studies conducted during the pilot implementation period demonstrated favorable outcomes among program participants. Reported benefits included reductions in anemia prevalence and low birth weight incidence, improvements in dietary intake and nutritional status, and increased nutrition knowledge and healthy dietary behaviors among pregnant women, lactating women, and young children [63]. Furthermore, the integrated provision of supplementary foods, nutrition education, individualized counseling, and regular nutritional assessment was shown to be more effective than nutrition education alone in addressing nutritional risks among vulnerable populations [63]. These findings provided important evidence supporting the nationwide expansion and institutionalization of the Nutrition Plus Program within the Korean public health system [63].

Ultimately, the Nutrition Plus Program represents an example of adapting and further developing the WIC model within the Korean context and has played an important role in reducing nutritional disparities and improving health equity among vulnerable populations. Furthermore, the program demonstrates the expansion of Korean nutrition policy from universal dietary improvement initiatives toward targeted public nutrition policies focused on identifying and managing nutritional risks among vulnerable groups. These policy experiences may also have important implications as models for maternal and child nutrition and community-based nutrition interventions applicable to future Official Development Assistance (ODA) programs in developing countries.

## 8. The 2010s

The 2010s can be characterized as a “health equity enhancement phase” during which the concept of health equity became actively incorporated into national policy and nutrition policy expanded from universal approaches toward interventions targeting vulnerable populations. During this period, nutrition policy increasingly functioned not only as a health promotion strategy but also as a public policy instrument aimed at reducing nutritional vulnerability and social inequalities.

### 8.1. National Health Plan 2020 (HP2020)

Beginning in the 2010s, Korean nutrition and health promotion policies expanded beyond simple lifestyle modification toward approaches emphasizing health equity and support for vulnerable populations [64,65]. In particular, the National Health Plan 2020 (HP2020) functioned as a comprehensive national strategy integrating life-course health management and healthy lifestyle promotion, including nutrition, physical activity, and smoking cessation. The plan established the extension of healthy life expectancy and reduction in health disparities as major national goals and identified healthy lifestyle practices, including nutrition, as key policy areas [64,65].

A major policy shift occurred following partial revisions to the National Health Promotion Act in March 2014. Specifically, Article 4, Clause 2–4 was added to require that National Health Plans include health promotion strategies for vulnerable populations such as children, women, older adults, and individuals with disabilities [66]. This revision reflected a transition from universal health promotion approaches toward customized policies considering socioeconomic vulnerability and health disparities among population groups. These legal and institutional changes directly influenced nutrition education policy. Nutrition education programs implemented through schools, communities, and public health centers expanded beyond simple information delivery toward policy interventions addressing nutritional imbalance and improving food accessibility among vulnerable populations. In addition, strengthened local government-based health promotion programs facilitated the expansion of community nutrition education initiatives and individualized nutrition management services tailored to different life stages and target populations [66].

HP2020 contributed to institutionalizing health equity within Korean nutrition policy by strengthening nutrition interventions for nutritionally vulnerable populations and expanding community-based nutrition services. Building upon earlier initiatives such as the Nutrition Plus Program, nutrition policies increasingly targeted low-income households, pregnant women, infants, older adults, and other high-risk groups through life-course nutrition management and community-based support programs [67,68,69]. As a result, health equity became an important principle guiding national nutrition policy, and nutrition interventions were increasingly integrated into broader health promotion and community health strategies [67,68,69]. However, policy evaluations suggested that continued efforts were required to further reduce socioeconomic disparities in nutritional health outcomes and improve access to nutrition services among vulnerable populations [68,69].

Consequently, HP2020 represented an important milestone in the evolution of Korean nutrition policy from a focus on individual dietary behavior change toward a national strategy emphasizing health equity and protection of vulnerable populations.

### 8.2. National Nutrition Management Act

As Korean nutrition policy evolved from traditional nutrition improvement and education approaches toward more systematic and integrated management systems, the National Nutrition Management Act was enacted in 2010 [70]. Whereas the Food Sanitation Act had primarily focused on protecting public health through food safety and hygiene management, the National Nutrition Management Act established a broader and more proactive legal framework aimed at improving nutritional status and preventing chronic diseases. This shift reflected the changing focus of Korean nutrition policy from addressing nutritional deficiencies toward preventing chronic diseases closely associated with dietary behaviors, including obesity, diabetes, and hypertension [71].

The National Nutrition Management Act included provisions related to life-course nutrition management, nutrition support for vulnerable populations, nutrition surveys and research, and strengthened responsibilities for both national and local governments [70]. Through this legislation, nutrition policy gained greater legal and institutional authority beyond simple recommendations, particularly supporting the expansion of customized nutrition management programs targeting nutritionally vulnerable populations such as children, pregnant women, and older adults [72]. In addition, national surveillance systems such as KNHANES strengthened evidence-based policymaking by enabling continuous monitoring of dietary behaviors and nutritional status among the Korean population [52].

Currently, the National Nutrition Management Act functions as a core legal framework linked to the National Health Plan and serves as the legislative basis for various national nutrition programs [71]. In particular, as Korea continues to experience low birth rates, population aging, and increasing chronic disease burdens, the importance of life-course nutrition management and vulnerable population-centered policies has become increasingly emphasized [72]. Ultimately, the National Nutrition Management Act provided a critical institutional foundation for the transition of Korean nutrition policy from education- and awareness-centered approaches toward an integrated national nutrition management system based on governmental responsibility.

### 8.3. Centers for Children’s and Social Welfare Foodservice Management

The Center for Children’s Foodservice Management was introduced based on the Special Act on Safety Management of Children’s Dietary Life, which was enacted in 2009. Pilot projects were subsequently implemented as part of the policy development process. In 2010, pilot centers were established in selected regions, and full-scale nationwide installation and operation began in 2011 [73,74]. Following confirmation of the program’s effectiveness, the centers were expanded nationwide and have become one of the representative community-based nutrition management support systems operated by local governments in Korea [75].

The centers were introduced as part of a national policy initiative aimed at systematically improving foodservice hygiene and nutrition management for infants and young children. During the late 2000s, the increasing number of dual-income households and expanded use of childcare facilities increased the importance of meals provided in childcare centers and kindergartens. However, many small-scale foodservice facilities lacked registered dietitians, creating substantial gaps in hygiene and nutrition management [76]. In response, the government introduced the Center for Children’s Foodservice Management system to improve the quality of foodservice and establish safer dietary environments for young children.

The centers were designed based on research and policy development initiatives led by the Korea Food and Drug Administration and included the development of food safety and nutrition education programs for children as well as standardized foodservice management systems [77]. The centers provided hygiene and safety management support, menu planning, nutrition education, and training programs for foodservice personnel in small-scale childcare facilities and kindergartens, thereby supplementing previously insufficient field-based nutrition management systems. This model can be regarded as a Korean-style community foodservice management system that enables professional nutrition services to be provided even in facilities without full-time dietitians.

The introduction of these centers has been evaluated positively in several respects. First, the overall hygiene and safety standards of meals provided to children improved substantially, and standardized menu planning and nutrition management became possible [76]. Second, nutrition education programs improved dietary awareness not only among children but also among parents and teachers within local communities [77]. Third, the expanded roles of dietitians and foodservice workers strengthened community-based nutrition management systems. Consequently, the Center for Children’s Foodservice Management has been recognized as an integrated public nutrition service model combining food safety, nutrition management, education, and field-based consultation.

Based on operational experiences and program outcomes, the functions and target populations of the centers were subsequently expanded. As the need for foodservice management among broader vulnerable populations emerged, the system evolved into the “Centers for Children’s and Social Welfare Foodservice Management,” which also support older adults, individuals with disabilities, and users of social welfare facilities [78]. This expansion reflects the government’s commitment to systematically managing nutrition improvement and health promotion among vulnerable populations at the national level.

In particular, the Centers for Social Welfare Foodservice Management have continuously improved foodservice quality through enhanced evaluation systems, expanded stakeholder participation, and strengthened professional capacity [78]. Through these efforts, the centers have contributed to improving the health status of populations vulnerable to nutritional imbalance. Overall, the introduction and expansion of these centers represent a major example of how Korean nutrition policy has evolved beyond food safety management toward life-course and population-specific nutrition management systems.

## 9. The 2020s

The 2020s can be characterized as a “precision nutrition transition phase” during which nutrition policy has increasingly shifted from population-average approaches toward personalized nutrition management driven by advances in digital technology and growing public interest in health. Korea, which previously developed its nutrition policies through support from the international community, has now transitioned beyond self-sufficiency toward sharing accumulated policy experiences, institutional systems, and educational models with developing countries through Official Development Assistance (ODA). In this context, future nutrition policy should expand beyond domestic health management toward contributing to global public health while simultaneously advancing toward precision nutrition systems that integrate individual health conditions, lifestyle behaviors, food environments, and digital health data.

### 9.1. National Health Plan 2030 (HP2030)

Health Plan 2030 (HP2030) serves as a bridge connecting international health agendas, including the United Nations Sustainable Development Goals (SDGs) and WHO health targets, with domestic Korean health policy [69]. The development of HP2030 involved not only the Ministry of Health and Welfare and the Korea Disease Control and Prevention Agency but also multiple governmental ministries, including the Ministry of Employment and Labor, Ministry of Gender Equality and Family, Ministry of National Defense, Ministry of Education, and Ministry of Science and ICT. Through this interministerial collaboration, health promotion was positioned as a comprehensive national policy agenda integrated across governmental sectors.

Within HP2030, nutrition policy remained a core component of healthy lifestyle promotion and was reorganized around food safety, healthy dietary practices, and improved accessibility to nutrition services [72]. In particular, the plan expanded beyond individual-centered dietary improvement approaches by incorporating nutrition disparities according to income level as a major monitoring indicator and identifying health equity improvement as a key policy objective [72]. In addition, HP2030 strengthened policy approaches linking dietary behaviors with the prevention and management of noncommunicable diseases [79]. These developments demonstrate that Korean nutrition policy has evolved beyond simple dietary guidance into a national strategy aimed at reducing health disparities and preventing chronic diseases.

However, recent evaluations have shown worsening trends in several major nutrition indicators, including decreased fruit, vegetable, and calcium intake and reduced rates of healthy dietary practice [72]. These trends are closely associated with increasing prevalence of obesity, hypertension, and diabetes. The decline in fruit and vegetable consumption may be partially attributable to the growing reliance on processed and convenience foods, increased frequency of eating outside the home, and changes in dietary patterns characterized by higher consumption of energy-dense foods and sugar-sweetened beverages. Similarly, decreasing calcium intake has been linked to reduced consumption of milk and dairy products, particularly among adolescents and young adults, as well as broader shifts toward irregular eating habits and meal skipping. These dietary transitions reflect the rapid changes in Korea’s food environment accompanying economic development, urbanization, and lifestyle modernization [80].

Furthermore, healthy life expectancy has recently declined, while the gap between overall life expectancy and healthy life expectancy has widened, indicating a reduction in the period of healthy living among the population [81]. As population aging accelerates, complex problems involving inadequate nutrient intake, functional decline, sarcopenia, and chronic disease burdens among older adults have become increasingly significant. Consequently, Korea’s low birth rate and aging society require more differentiated nutrition policies considering individual health conditions and living environments rather than relying solely on population-average recommendations.

This need is also reflected in the recently revised 2025 Dietary Reference Intakes for Koreans (KDRIs 2025), which were developed in response to rapid population aging, increasing chronic disease burden, changing dietary environments, and accumulating scientific evidence regarding life-course nutrition and disease prevention [80]. The KDRIs 2025 emphasize the importance of balanced dietary patterns, limiting excessive intake of sugars and sodium, addressing nutritional challenges associated with aging, and providing more evidence-based recommendations tailored to different life stages and physiological conditions [80]. In addition, the revision highlights the necessity of strengthening nutrition policies that support healthy aging, chronic disease prevention, and improved quality of life throughout the lifespan [80].

Future nutrition policy should therefore move beyond approaches limited to improving individual dietary habits and strengthen structural interventions such as improving food environments, creating health-supportive settings, and expanding targeted nutrition support for vulnerable populations. Moreover, increasing interest in personalized nutrition highlights the need for precision nutrition policies integrating genomic information, health status, lifestyle factors, and individual risk profiles. Consistent with the direction of KDRIs 2025, future nutrition policies should incorporate advances in nutrition science, digital health technologies, and individualized nutrition assessment while maintaining population-wide public health approaches [80]. However, HP2030 remains largely focused on population-level targets and behavioral interventions, with relatively limited consideration of life-course nutritional needs, healthy aging, and individualized nutrition management. As Korea faces rapid population aging, increasing chronic disease burden, and widening health disparities, nutrition policy will need to evolve beyond traditional population-based approaches. Accordingly, future Korean nutrition policy should integrate personalized nutrition, digital health technologies, life-course nutrition management, healthy aging strategies, and health equity considerations into a more comprehensive precision nutrition framework while preserving the strengths of universal public health interventions [80,82].

### 9.2. International Development Cooperation Programs

Korea has increasingly expanded its accumulated experiences in nutrition policy and nutrition education into ODA programs targeting developing countries [83,84,85,86,87]. In particular, with support from the Korea International Cooperation Agency (KOICA), projects have been implemented to systematize Korea’s historical experiences in nutrition policy development and adapt these institutional models to the sociocultural contexts of developing countries [7,84].

Korea has substantial experience addressing the double burden of malnutrition through national policies and educational systems. These experiences have evolved into structured approaches involving community-based nutrition education, the dietitian system, and life-course nutrition management policies [64]. Consequently, the Korean nutrition model has been applied to ODA programs not merely as knowledge transfer but as an integrated framework incorporating institutions, professional workforce development, educational content, and community-based implementation systems.

A distinctive characteristic of these ODA initiatives is their adoption of a “system transfer” approach rather than focusing solely on material assistance or short-term education programs [85,86,87]. Based on Korean nutrition policy experiences, these projects integrated policy design, workforce training, and educational content development into transferable systems capable of long-term local operation. In particular, these initiatives identified inadequate nutrition awareness and insufficient education—along with limited food accessibility—as major contributors to nutritional problems and therefore emphasized sustainable community-based education and local workforce development.

This approach can be observed in nutrition projects implemented in Mongolia and Cambodia by WITH (Wholistic Interest Through Health), a Korean food and nutrition-focused Non-Governmental Organization (NGO) [86]. The Mongolian nutrition improvement project, implemented in the late 2000s, established a community-based nutrition education system to address micronutrient deficiencies among women and children [86]. Local health workers and teachers were trained as educators, and educational materials were developed by adapting Korea’s life-course nutrition management concepts to Mongolia’s food culture and food accessibility conditions [83]. Practical cooking sessions and meal-planning education were also incorporated to promote behavioral changes in dietary practices [86].

Similarly, maternal and child health and nutrition projects implemented in Cambodia focused on nutrition management for infants, young children, and pregnant women [86]. Community health workers served as key educational personnel delivering programs related to breastfeeding, complementary feeding, hygiene management, and balanced diets [86]. In addition, supplementary foods and recipes using locally available ingredients were developed and integrated into cooking practice sessions to facilitate practical dietary improvements. These projects represent examples of how Korea’s life-course nutrition policy and community-based nutrition education experiences were adapted to local contexts [86].

The Pakistan project initiated in 2020 represents a more systematic application of the Korean nutrition ODA model [87]. During the preparatory stage, Korean experts and local personnel collaboratively established the design framework for the nutrition education system while emphasizing localization strategies reflecting Pakistan’s health and educational infrastructure. Subsequently, local program managers participated in intensive training programs in Korea covering nutrition policy, dietitian management systems, and community nutrition management structures. Based on this training, educational modules and teaching materials were jointly developed. During implementation, the Pakistan–Korea Nutrition Center (PKNC) established a multilayered educational delivery system connecting Korean experts-master trainers-community educators-local residents. In addition, foods and recipes utilizing local ingredients were developed to integrate nutrition education with practical dietary improvement.

These cases from Mongolia, Cambodia, and Pakistan demonstrate how Korean experiences in nutrition policy and nutrition education can be adapted and disseminated through ODA programs in diverse ways. All three projects shared common strategies emphasizing education-centered approaches, system transfer, multilayered workforce development, and localization. By integrating educational interventions with food-based approaches, these projects promoted meaningful dietary improvements while supporting the establishment of sustainable local nutrition systems. Consequently, these approaches may provide valuable models for future nutrition improvement programs in developing countries.

## 10. Conclusions and Discussion

**Figure 3:**
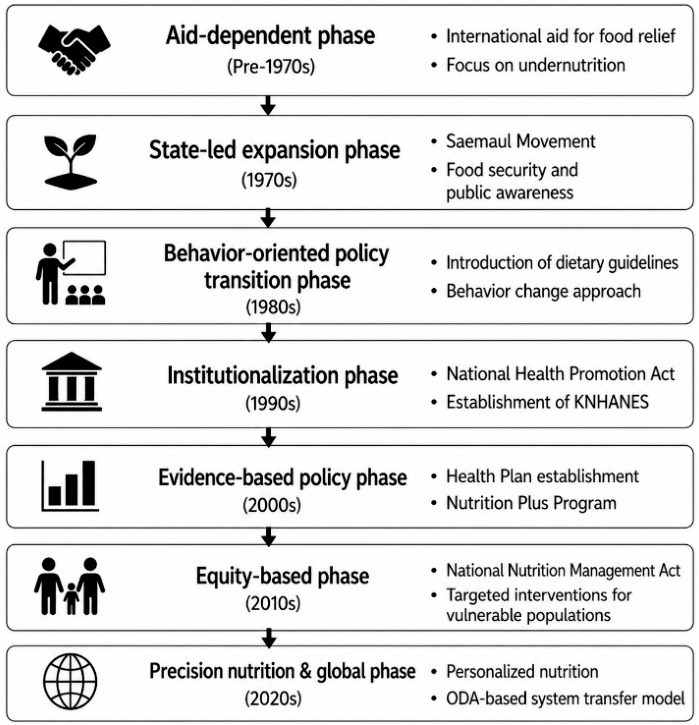
The Evolution phase of nutrition policy in South Korea from the 1960s to the 2020s.

This study examined the dynamic historical evolution of nutrition policy and nutrition education in Korea since the 1960s. The development of Korean nutrition policy can be categorized into several major phases according to socioeconomic changes, epidemiological transitions, and shifts in public health priorities, as summarized in Figure 3 and Table 7. The modern history of Korean nutrition policy represents a globally distinctive example of a country transitioning from an aid recipient to a donor country. During the postwar recovery period and throughout the 1960s and 1970s, Korea received extensive international assistance from organizations such as UNICEF, FAO, and WHO to address severe food shortages and undernutrition. Food aid, including milk powder, wheat flour, and corn products, played a critical role in supporting the survival and health of vulnerable populations. However, Korea did not rely solely on aid consumption; instead, it utilized these resources to establish self-sustaining national nutrition management infrastructures, including state-led dietary improvement campaigns, nutrition education systems, school foodservice programs, the dietitian system, and national nutrition surveys. As a result, Korea achieved food and nutrition self-sufficiency within a relatively short period and has now transitioned into a donor country contributing to nutrition improvement and health capacity building in developing countries through international development cooperation and Official Development Assistance (ODA).

Korea also experienced the challenges of nutrition transition associated with rapid economic growth and the westernization of dietary culture, resulting in increasing rates of overnutrition and chronic diseases. At the same time, however, Korea developed distinctive response strategies based on traditional dietary culture. Through collaboration among the government, local authorities, academic institutions, and professional organizations such as the Korean Dietetic Association, traditional Korean dietary patterns emphasizing vegetables and fermented foods were incorporated into modern public health policies. In addition, dietary improvement programs utilizing local agricultural products and traditional foods were combined with continuous nutrition education initiatives. These approaches contributed to the development of a Korean nutrition policy model integrating traditional dietary culture with modern nutrition management and chronic disease prevention strategies.

To provide a broader context for interpreting the evolution of Korean nutrition policy, Table 8 presents representative socioeconomic and health indicators that accompanied each major policy phase. During the aid-dependent phase, Korea experienced severe economic hardship, with GDP per capita of only USD 158.8 in 1960, accompanied by a high infant mortality rate of 74 deaths per 1000 live births [88,89]. Over subsequent decades, GDP per capita increased substantially to USD 33,645.8 in 2020, while infant mortality declined to 3 deaths per 1000 live births and life expectancy increased from 62.3 years in 1970 to 83.5 years in 2020 [88,89,90]. These improvements occurred alongside the progressive development of nutrition-related institutions and programs, including nutrition education, school meal services, national nutrition surveillance systems, and targeted nutrition support programs [15,16,26,30,51,62,63,64,65]. Although these favorable trends cannot be attributed solely to nutrition policies, they suggest that nutrition interventions were implemented within a broader context of rapid socioeconomic development and contributed to improvements in population health and nutritional well-being [7,8,88,89,90]. The simultaneous improvement in economic, mortality, and longevity indicators suggests that nutrition policies evolved in parallel with broader public health, educational, and social development efforts, highlighting the importance of multisectoral approaches to nutrition governance and reflecting the broader nutrition transition associated with rapid socioeconomic development [9,10,11,12]. Therefore, the achievements observed during each policy phase should be interpreted within the broader context of socioeconomic development, public health advancement, and educational expansion rather than being attributed solely to nutrition policy interventions.

At the same time, the findings highlight the changing nature of nutrition challenges across policy phases. Early policies primarily focused on addressing food insecurity, undernutrition, and micronutrient deficiencies, whereas later policies increasingly emphasized chronic disease prevention, health equity, and life-course nutrition management [51,66,67,68,69,70,71,72,73,74,75,76,77]. This transition reflects the epidemiological and nutrition transitions experienced by Korea during rapid industrialization and urbanization [9,10,11,12]. Notably, despite substantial economic growth and improvements in traditional health indicators, recent national evaluations have reported declines in fruit, vegetable, and calcium intake as well as decreases in healthy dietary practice rates [78,83]. These findings suggest that future nutrition policies should move beyond ensuring food availability and continue to address diet quality, health equity, and sustainable dietary behaviors in an increasingly complex food environment [75,77,83]. Beyond these domestic developments, comparison with other Asian countries further highlights the distinctive trajectory of Korean nutrition policy development. Japan is often regarded as one of the earliest countries in Asia to establish a comprehensive nutrition policy system. Previous studies have emphasized that Japan’s success was supported by the early institutionalization of nutrition science, national nutrition surveys, dietitian training systems, and school meal programs [91,92,93,94,95,96]. Although Japan experienced severe food shortages following World War II, many nutrition-related institutions and research systems had already been established before the war, and subsequent nutrition policies were largely developed through the reconstruction and expansion of existing systems [91,92]. In contrast, China’s nutrition policy has been characterized by large-scale government-led programs targeting undernutrition and regional disparities, particularly among rural populations and children [96]. Major initiatives such as the Nutrition Improvement Program for Rural Compulsory Education Students and the National Nutrition Plan (2017–2030) focused on improving nutritional status through nationwide interventions implemented under strong central government leadership [96]. Taiwan has followed a somewhat different path by emphasizing food and agricultural education. The Food and Agricultural Education Act enacted in 2022 established a national framework integrating nutrition, agriculture, food culture, environmental sustainability, and local food systems, thereby expanding nutrition policy beyond conventional health promotion approaches [95]. Taiwan has also strengthened nutrition standards for school meals and incorporated nutrition education into school-based health promotion programs [94].

While Korea shares certain features with each of these countries, its experience demonstrates several distinctive characteristics. Unlike Japan, Korea developed most of its nutrition-related institutions after the Korean War while simultaneously undergoing rapid industrialization, urbanization, and socioeconomic transformation. Unlike China, which primarily relied on large-scale centralized nutrition programs, Korea gradually institutionalized nutrition governance through the establishment of the dietitian system, national nutrition surveys, school meal programs, community nutrition services, and targeted nutrition support programs for vulnerable populations. Furthermore, unlike Taiwan, whose recent policies have largely emphasized food and agricultural education, Korean nutrition policy evolved into a comprehensive life-course nutrition management system encompassing maternal, infant, child, adolescent, adult, and older adult populations. These experiences suggest that the Korean case is characterized by the rapid institutionalization of nutrition policy during a compressed period of socioeconomic development rather than by any single policy intervention [91,92,93,94,95,96].

The Korean experience provides important implications for developing countries facing the double burden of malnutrition characterized by the coexistence of undernutrition and overnutrition. In particular, Korea’s ability to transform external assistance into a foundation for self-sustaining national nutrition policy and to integrate traditional dietary culture with modern health policy represents a valuable model for sustainable nutrition improvement. Accordingly, international development cooperation programs based on Korean nutrition policy and education experiences may contribute not only to technical transfer but also to the development of context-specific health promotion strategies that consider local culture, food accessibility, health systems, and workforce capacity.

The historical development of Korean nutrition policy examined in this study also provides several important policy implications. First, sustainable nutrition policy requires not only food provision or nutrition education but also the simultaneous establishment of legal systems, professional workforce capacity, surveillance systems, and community-based delivery structures. Second, in response to changing dietary patterns and increasing chronic disease burdens, nutrition policy should move beyond universal population-based recommendations and incorporate customized approaches considering life stage, socioeconomic status, health conditions, and food environments. Third, nutrition policy can expand beyond domestic health promotion strategies to function as an international cooperation model supporting capacity building and sustainable dietary improvement in developing countries.

Nevertheless, this study has several limitations. Although it aimed to comprehensively examine the overall development of Korean nutrition policy and education, not all policies and programs could be included because of limitations in data accessibility. In particular, detailed analyses of local government-based nutrition programs implemented after the 2000s were limited. Furthermore, although major national programs such as the National Health Plan, Nutrition Plus Program, and Centers for Children’s and Social Welfare Foodservice Management were reviewed, this study could not fully examine program effectiveness, regional differences, or population-specific outcomes in depth.

Despite these limitations, this study is meaningful because it systematically collected and integrated diverse historical materials related to Korean nutrition policy and nutrition education, particularly for the period before the 2000s, and presented them within a unified developmental framework. In particular, this study has academic and policy significance because it integrated fragmented policy documents and research findings and interpreted them not only within the domestic Korean context but also from the perspective of international development cooperation and ODA. Future studies incorporating detailed analyses of local government nutrition policies, evaluations of long-term national programs, assessments of nutrition policy outcomes among vulnerable populations, and applications of digital nutrition management systems may further clarify the scalability and international applicability of the Korean nutrition policy model.

Overall, this study aimed to present the historical development of Korean nutrition policy not merely as a national case study but as a practical strategic model for global health and nutrition improvement. Korea’s experience can be understood as a continuous transition from an aid-recipient country toward a comprehensive nutrition policy system encompassing food security, nutrition education, legislation and institutionalization, evidence-based policymaking, equity-oriented support, precision nutrition, and international development cooperation. Future Korean nutrition policy should continue evolving to address changing chronic disease patterns, population aging, health disparities, and transformations in food environments while integrating personalized nutrition, digital health, precision nutrition, and sustainable dietary practices. Furthermore, the globalization of Korean nutrition policy should not be pursued simply as policy exportation but rather as a form of mutual learning-based international cooperation that respects local cultural and institutional contexts. Through such approaches, Korea may contribute more responsibly to global nutrition improvement and health equity promotion.

## Figures and Tables

**Table 1:** National Healthy Lifestyle Guidelines (1984) [20].

1. Maintain personal hygiene: wash hands before eating and brush teeth afterwards. 2. Eat a variety of foods, and choose a diet moderate in salt. 3. Take regular vaccinations and health checkups. 4. Stop smoking and do not drink too much. 5. Exercise regularly and maintain physical vitality. 6. Enjoy working and life. 7. Maintain public discipline and safety precautions. 8. Do not waste resources and maintain a healthy environment.

**Table 2:** Dietary Guidelines for Koreans (1986) [20,42].

1. Eat a variety of foods in balanced proportions. 2. Maintain a healthy body weight. 3. Consume adequate amounts of protein. 4. Limit fat intake to approximately 20% of total energy intake. 5. Drink milk daily. 6. Avoid eating excessively salty foods. 7. Maintain good dental health. 8. Moderate the consumption of alcohol, tobacco, and caffeinated beverages. 9. Maintain balance between dietary habits and daily life. 10. Enjoy meals pleasantly.

**Table 3:** National Dietary Guidelines (1990) [20].

1. Eat a variety of food, with recommended fat intake equaling to or less than 20% total calories. 2. Maintain ideal body weight and prevent obesity. 3. Eat food low in salt, Salt intake should not exceed 10 g. 4. Do not drink too much. 5. Eat regularly and enjoy meals.

**Table 4:** The First phase of National Health Plan 2010 (HP2010) [57,58].

Goals in the Nutrition Sector	
1. Increase the proportion of the population consuming nutrients within the recommended intake range for nutrients of concern from 30% to 50%. 2. Increase the proportion of the population maintaining a healthy body weight (18.5 ≤ body mass index < 25) from 68.7% to 75%.	
Major subprogram	Subprogram Description
Development and dissemination of dietary guidelines for obesity reduction and the prevention of diabetes and hypertension.	1. Establishment of national dietary goals.
	2. Development and dissemination of life cycle–specific dietary guidelines.
	3. Development of dietary guidelines for obesity reduction and the prevention of chronic diseases such as diabetes and hypertension.
Provision of nutrition management services for social welfare facilities and childcare centers.	1. Assessment of community nutrition improvement programs at public health centers and establishment of program priorities.
	2. Assessment of the current status and identification of problems in foodservice management at social welfare facilities and childcare centers.
	3. Establishment of foodservice management and support strategies for facility users and young children.
	4. Establishment of an effective nutrition program system for public health centers.
Institutionalization of nutrition labeling for processed foods.	1. Development of strategies for advancing the nutrition labeling system: investigation of the current status of nutrition labeling, identification of existing problems, and collection of opinions from consumers, industry representatives, and experts.
	2. Establishment of educational and promotional strategies for the consumer nutrition labeling system and development of a delivery system.
	3. Establishment and support of a nutrition labeling support system for food industries.
	4. Provision of nutrition labeling information and establishment of a national nutrition information system.

**Table 5:** The second phase of National Health Plan 2010 (HP2010) [57,58].

Goals
1. Expansion of nutrition support programs for vulnerable populations. 2. Development and dissemination of educational materials for healthy dietary practices. 3. Institutionalization of nutrition labeling for processed foods. 4. Establishment of a national nutrition information production and dissemination system. 5. Development and dissemination of nutrient-dense recipes and cooking methods for older adults.
Nutrition subprograms Description
A. Adequate Intake of Micronutrients
a. Increase the proportion of the population consuming calcium at adequate levels. b. Increase the proportion of the population consuming iron at adequate levels. c. Increase the proportion of the population consuming vitamin A at adequate levels. d. Increase the proportion of the population consuming riboflavin at adequate levels.
B. Prevention and Management of Chronic Diseases
a. Increase the proportion of the population whose fat intake falls within the Acceptable Macronutrient Distribution Range (AMDR). b. Increase the proportion of the population aged ≥6 years consuming less than 2000 mg of sodium per day. c. Increase the proportion of the population aged ≥6 years consuming at least 500 g of fruits and vegetables per day.
C. Weight Management
a. Increase the proportion of adults maintaining a healthy body weight (18.5 ≤ BMI < 25). b. Reduce the proportion of underweight adults.
D. Life-Course Nutrition Management
a. Increase the proportion of infants exclusively breastfed for the first 6 months of life. b. Reduce the prevalence of breakfast skipping caused by unhealthy dietary habits. c. Reduce the proportion of older adults aged ≥ 65 years with insufficient nutrient intake. d. Reduce the proportion of women of reproductive age (10–49 years) with iron-deficiency anemia.
E. Food Safety and Nutrition Services
a. Increase the proportion of households with food security. b. Increase participation in dietary support programs, including food assistance, WIC, and school meal programs. c. Increase the proportion of the population receiving nutrition management services, including nutrition education and counseling. d. Increase the proportion of the population using nutrition labeling information when selecting foods.

**Table 6:** Dietary Goals and Guidelines for Koreans [59].

Dietary Goals for Koreans
1. Consume energy and protein at recommended levels. 2. Increase the intake of calcium, iron, vitamin A, and riboflavin. 3. Limit fat intake to less than 20% of total energy intake. 4. Limit salt intake to less than 10 g per day. 5. Reduce alcohol consumption. 6. Maintain a healthy body weight (18.5 ≤ BMI < 25). 7. Maintain healthy eating habits. 8. Promote and develop the traditional Korean diet. 9. Ensure hygienic food handling and management. 10. Reduce food waste.
Dietary Guidelines for Koreans
1. Consume a variety of foods, including grains, vegetables, fruits, meat and fish, and dairy products. 2. Avoid salty foods and eat a less salty diet. 3. Increase physical activity and consume appropriate amounts of food to maintain a healthy body weight. 4. Enjoy meals and do not skip breakfast. 5. Limit alcohol consumption. 6. Prepare food hygienically and only in necessary amounts. 7. Enjoy the traditional Korean diet centered on rice as the staple food.

**Table 7:** The Evolution of Nutrition Policy in South Korea: Policy Drivers, Achievements, and Remaining Challenges from the 1960s to the 2020s.

Period	Principal Policies and Programs	Underlying Factors for Introduction	Major Achievements	Remaining Challenges and Limitations
Aid-dependent phase (Pre-1970s)	International food aid programs, Applied Nutrition Program (ANP), establishment of dietitian licensing system	Post-war food shortages, widespread undernutrition, dependence on international assistance, national reconstruction efforts	Established the foundation of Korea’s modern nutrition system through nutrition education, food assistance, and professional workforce development	Continued dependence on foreign aid and limited domestic capacity for food production and nutrition services
State-led expansion phase (1970s)	Saemaul Movement, mixed-grain campaign, rice-saving campaign, National Nutrition Improvement initiatives	Rapid industrialization, food shortages, rice supply constraints, state-led economic development strategy	Improved food availability and nutrition awareness while integrating nutrition into national development policies	Policy emphasis remained focused on food security and calorie sufficiency rather than dietary quality and chronic disease prevention
Behavior-oriented policy transition phase (1980s)	School Meals Act, Dietary Guidelines for Koreans, school-based nutrition education	Economic growth, urbanization, diversification of food consumption patterns, increasing public interest in health	Expanded nutrition education and established a healthier school food environment through institutional school meal services	Increasing consumption of processed foods and dietary shifts associated with nutrition transition
Institutionalization phase (1990s)	National Health Promotion Act, KNHANES, expansion of dietitian workforce and public nutrition programs	Democratization, expansion of public health systems, growing burden of chronic diseases	Established institutional and surveillance systems supporting evidence-based nutrition policymaking	Emerging obesity and chronic disease burden associated with rapid lifestyle changes
Evidence-based policy phase (2000s)	Health Plan 2010/2020, Nutrition Plus Program, Childcare Foodservice Management Support Centers	Growing demand for evidence-based public health policies, increasing healthcare costs, population aging	Strengthened nutrition support for vulnerable populations and expanded community-based nutrition management systems	Persistent socioeconomic disparities in dietary quality and increasing prevalence of obesity-related conditions
Equity-based phase (2010s)	National Nutrition Management Act, community nutrition programs, nutrition services for vulnerable populations	Growing concern regarding health equity, rapid population aging, widening health disparities	Strengthened legal and institutional frameworks for nutrition management and expanded support for nutritionally vulnerable groups	Continued inequalities in nutritional status and health outcomes among socioeconomic groups
Precision nutrition and global phase (2020s–present)	HP2030, KDRIs 2025, personalized nutrition initiatives, digital health approaches, international nutrition cooperation programs	Population aging, low fertility, digital transformation, increasing interest in precision nutrition and global health cooperation	Expanded life-course and equity-oriented nutrition strategies while promoting international cooperation and future-oriented nutrition policies	Recent evaluations have reported declines in fruit, vegetable, and calcium intake as well as healthy dietary practice rates, indicating ongoing challenges in improving diet quality despite policy advancement

**Table 8:** Evolution of Korean Nutrition Policy and Changes in Key Socioeconomic and Health Indicators from the 1960 to the 2020.

Policy Phase	Representative Year	Principal Policies and Programs	GDP per Capita ^1^ (USD)	Infant Mortality ^1^ (Per 1000 Live Births)	Life Expectancy ^2^ (Years)
Aid-dependent phase	1960	International food aid programs, Applied Nutrition Program (ANP), establishment of dietitian licensing system	158.8	74	N/A ^3^
State-led expansion phase	1970	Saemaul Movement, mixed-grain campaign, rice-saving campaign, National Nutrition Improvement initiatives	281.8	45	62.3
Behavior-oriented policy transition phase	1980	School Meals Act, Dietary Guidelines for Koreans, school-based nutrition education	1745.6	28	66.1
Institutionalization phase	1990	National Health Promotion Act, KNHANES, expansion of dietitian workforce and public nutrition programs	6812.9	12	71.7
Evidence-based policy phase	2000	Health Plan 2010/2020, Nutrition Plus Program, Childcare Foodservice Management Support Centers	12,710.3	6	76.0
Equity-based phase	2010	National Nutrition Management Act, community nutrition programs, nutrition services for vulnerable populations	24,071.3	3	80.2
Precision nutrition and global phase	2020	HP2030, KDRIs 2025, personalized nutrition initiatives, digital health approaches, international nutrition cooperation programs	33,645.8	3	83.5

Representative year values were selected to reflect the socioeconomic and health conditions corresponding to each major phase of Korean nutrition policy development. ^1^ GDP per capita and infant mortality data were obtained from the World Bank World Development Indicators [88,89]. ^2^ Life expectancy data were obtained from the Statistics Korea Life Tables (Korean Statistical Information Service, KOSIS) [90]. ^3^ Life expectancy data for the pre-1970 period were not available from KOSIS; therefore, the value for 1960 was not reported.

## Data Availability

No new data were created or analyzed in this study. Data sharing is not applicable to this article.

## Abbreviations

The following abbreviations are used in this manuscript:

ANP	Applied Nutrition Project
AI	Artificial Intelligence
BCG	Bacillus Calmette–Guérin
CARE	Cooperative for American Relief Everywhere
DPT	Diphtheria, Pertussis, and Tetanus
FAO	Food and Agriculture Organization
GDP	Gross Domestic Product
HP	Health Plan
KBS	Korean Broadcasting System
KDRI	Dietary Reference Intakes for Koreans
KNHANES	Korea National Health and Nutrition Examination Survey
KOICA	Korea International Cooperation Agency
KOSIS	Korean Statistical Information Service
KRDA	Korea Recommended Dietary Allowances
NGO	Non-Governmental Organization
ODA	Official Development Assistance
PKNC	Pakistan–Korea Nutrition Center
SDGs	Sustainable Development Goals
SUN	Surplus Utilization for Nutrition
UNICEF	United Nations Children’s Fund
UNCACK	United Nations Civil Assistance Command, Korea
WHO	World Health Organization
WIC	Special Supplemental Nutrition Program for Women, Infants, and Children
